# Trends in cervical cancer incidence and mortality in Mongolia, 2014–2024: a population-based analysis of pandemic-related disruptions

**DOI:** 10.3389/fonc.2026.1853617

**Published:** 2026-08-24

**Authors:** Nomin-Erdene Tsogtgerel, Dolgorkhand Adiyadorj, Gan-Erdene Ganbaatar, Amy Elizabeth Parry, María José González Méndez, You Lin Qiao

**Affiliations:** 1 School of Public Health, Dalian Medical University, Dalian, China; 2 Ministry of Health, Ulaanbaatar, Mongolia; 3 Center for Health Development, Ulaanbaatar, Mongolia; 4 National Centre for Epidemiology and Population Health, The Australian National University, Canberra, ACT, Australia; 5 Department of Primary Care and Family Health, Faculty of Medicine, University of Chile, Santiago, Chile; 6 School of Population Medicine and Public Health, Chinese Academy of Medical Sciences and Peking Union Medical College, Beijing, China

**Keywords:** cervical cancer, COVID-19, health system resilience, incidence, Mongolia, mortality

## Abstract

**Background:**

Cervical cancer remains a major public health concern in Mongolia, where screening coverage is estimated at approximately 38%, well below recommended levels, and access to timely diagnosis varies. This study examined temporal trends in incidence and mortality and assessed the impact of COVID-19–related disruptions on cancer detection.

**Methods:**

A nationwide population-based study was conducted using data from Mongolia’s national health information system (H-Info). All cervical cancer cases (ICD-10: C53.0-C53.9) and related deaths from 2014 to 2024 were included. Age-standardized incidence (ASIR) and mortality rates (ASMR) were calculated using the WHO world standard population as reference. Temporal trends were analyzed using log-linear and segmented regression models. The mortality-to-incidence ratio (MIR) was used as an ecological indicator of population-level cancer outcomes.

**Results:**

A total of 3,990 cases and 1,370 deaths were identified. Incidence increased between 2014 and 2016, declined thereafter, and dropped sharply during 2020–2022 (APC −29.4%), before rebounding in 2023–2024. In contrast, mortality declined gradually over the study period. MIR increased during the pandemic and declined following service recovery.

**Conclusions:**

Cervical cancer incidence in Mongolia is highly sensitive to disruptions in screening and diagnostic services. Divergent trends between incidence and mortality likely reflect reduced detection during the pandemic rather than true changes in disease burden. These findings highlight the importance of maintaining continuity of cancer screening and diagnostic services during health-system disruptions.

## 1 Introduction

Cervical cancer remains one of the most preventable malignancies, yet it continues to impose a substantial global burden, particularly in low- and middle-income countries (LMICs) where access to screening and timely treatment remains uneven (1, 2). Despite effective prevention through human papillomavirus (HPV) vaccination and screening, inequalities in access to early detection and treatment continue to drive variation in incidence and mortality across countries (3).

Mongolia illustrates many of these challenges. The health system operates within a tiered structure, where primary care facilities are responsible for screening, while diagnostic confirmation and treatment are largely concentrated in secondary and tertiary hospitals (4, 5). In practice, however, the country’s vast geographic landscape, low population density, and uneven distribution of health resources create substantial barriers to access, particularly for rural populations. Although a national cytology-based screening program has been in place since 2012, coverage remains relatively low, estimated at around 38%, which is well below the level required to achieve meaningful population-level impact. At the same time, rapid urbanization has concentrated demand for specialized services in Ulaanbaatar, increasing pressure on referral centers while leaving rural areas comparatively underserved.

Although previous research has described the burden of cervical cancer in Mongolia, there is limited evidence on how incidence and mortality have evolved over time, and even less on how these patterns are influenced by disruptions in health-service delivery. Importantly, routinely reported indicators such as incidence do not always reflect true changes in disease occurrence, rather, they may be strongly influenced by variations in screening and diagnostic activity. This makes interpretation of trends particularly challenging in settings where access to services is inconsistent.

The COVID-19 pandemic provides a valuable opportunity to better understand these dynamics. Across many countries, disruptions to screening programs and delays in diagnosis during the pandemic were associated with declines in reported cancer incidence, raising concerns about missed or delayed detection rather than real reductions in disease burden (3, 6–8). However, evidence from LMICs—where health systems may be more vulnerable to disruption—remains limited.

Against this background, this study aimed to assess temporal trends in cervical cancer incidence and mortality in Mongolia from 2014 to 2024 and to examine the impact of pandemic-related disruptions on cancer detection. By jointly analyzing incidence, mortality, and MIR, this study seeks to provide a more nuanced understanding of how routine cancer indicators reflect underlying health-system functioning and to inform strategies for strengthening cancer control in similar settings.

This study extends beyond descriptive trend analysis by integrating incidence, mortality, and the mortality-to-incidence ratio (MIR), providing a framework for interpreting cancer trends under conditions of health-system disruption.

## 2 Methods

### 2.1 Study design and data source

This nationwide population-based study used data from Mongolia’s Health Information System (H-Info), a centralized database that collects routine administrative and clinical information from health facilities across the country. The database includes patient demographics, diagnoses coded according to the International Classification of Diseases, 10th Revision (ICD-10), and mortality records.

All incident cervical cancer cases (ICD-10 codes C53.0–C53.9) and cervical cancer–related deaths (ICD-10 code C53 as the underlying cause of death) recorded between 1 January 2014 and 31 December 2024 were included. Coding practices remained consistent throughout the study period. Female population data were obtained from the National Statistics Office of Mongolia and used as denominators for calculating rates.

The dataset used in this study was provided by the Center for Health Development, Mongolia, as a pre-extracted, de-identified dataset for research purposes (Letter No. 1403; 24 December 2025). Data were accessed between 26 December 2025 and 15 February 2026. All personal identifiers were removed prior to data access. Ethical approval was obtained from the Medical Ethics Review Committee of the Ministry of Health, Mongolia (Approval No. 26/009; 25 February 2026), and the requirement for informed consent was waived due to the use of anonymized registry data. All methods were carried out in accordance with relevant guidelines and regulations. The authors did not have access to the full H-Info database and analyzed only the dataset provided for this study.

#### 2.1.1 Variables

Variables including age, residence (urban/rural), hospital type (primary, secondary, tertiary), and education level were extracted as recorded in the H-Info system. Residence was defined according to administrative classification within the database. Hospital type was categorized based on the national health system structure, and education level was recorded as reported in routine administrative records. These variables are routinely coded within the H-Info system using standardized administrative categories, and no additional coding schemes or derivations were applied by the authors.

#### 2.1.2 Data quality and cleaning

We assessed data completeness by examining the proportion of records with available data, as well as missing or unspecified values, for key variables using Microsoft Excel and R software (version 4.5.2).

A total of 3,990 cervical cancer cases were identified during the study period. Key variables, including age, residence, and hospital type, were fully recorded (100%), while education had minimal missing data (0.7%). The mortality dataset included 1,370 deaths, with date of death and age fully recorded (100%).

Data were screened for implausible values, including invalid age entries and inconsistent dates. No records were excluded based on data quality criteria. Missing data were not imputed, and analyses were conducted using available cases. Data completeness improved over time, with missing information more common in earlier years, likely reflecting the gradual strengthening of routine data recording systems.

#### 2.1.3 Measures

Age-standardized incidence rates (ASIR) and age-standardized mortality rates (ASMR) were calculated per 100,000 women using the direct method, with the WHO world standard population as reference. The mortality-to-incidence ratio (MIR) was calculated as the ratio of ASMR to ASIR and used as an ecological indicator of population-level cancer outcomes. MIR does not capture individual-level survival and should therefore be interpreted with caution.

### 2.2 Statistical analysis

Temporal trends in incidence and mortality were analyzed using log-linear regression models to estimate annual percent change (APC), calculated as 100 × (e^β − 1), where β represents the slope of the log-transformed rates over time.

Analyses were conducted for the overall study period (2014–2024), with additional descriptive comparisons across pre-pandemic (2014–2019), pandemic (2020–2022), and post-pandemic (2023–2024) periods. These periods were defined based on the timing of COVID-19–related disruptions to health services and subsequent recovery in Mongolia.

To explore potential changes in mortality trends, segmented (joinpoint) regression analysis was performed using the segmented package in R. Given the limited number of annual observations, models were restricted to a maximum of one joinpoint to avoid overfitting, with a minimum of three observations per segment. The location of the joinpoint was estimated directly from the data using an iterative model-fitting approach. Model selection was based on goodness-of-fit considerations and visual assessment of model fit. Changes in trends before and after the joinpoint were evaluated based on the estimated regression coefficients and their 95% confidence intervals.

APC estimates for the post-pandemic period (2023–2024) are based on a limited number of observations and should therefore be interpreted as descriptive rather than statistically robust.

Log-linear regression was selected as it provides a robust approach for modeling temporal trends in population-level rates under the assumption of constant relative change over time.

All analyses were conducted using R software (version 4.5.2), and a two-sided p-value < 0.05 was considered statistically significant.

All analyses and interpretations were conducted and verified by the authors.

## 3 Results

### 3.1 Overall cases and deaths

A total of 3,990 incident cervical cancer cases and 1,370 cervical cancer–related deaths were recorded in Mongolia between 2014 and 2024.

### 3.2 Trends in incidence and mortality

The female population of Mongolia ranged from 1,111,435 to 1,273,597 between 2014 and 2024, based on data from the National Statistics Office, Mongolia.

Age-standardized incidence rates (ASIR) showed substantial variation over time. ASIR increased from 30.5 per 100,000 women (95% CI: 17.7–43.2) in 2014 to a peak of 45.3 (30.0–60.6) in 2016, followed by a gradual decline to 27.6 (17.2–37.9) in 2019.

A marked reduction in incidence was observed during the COVID-19 period, with ASIR declining to 11.4 (5.6–17.2) in 2022, representing an approximate 75% decrease from the 2016 peak. This was followed by a sharp rebound to 38.7 (26.7–50.8) in 2023, with rates remaining stable in 2024 (39.5; 27.4–51.7), approaching—but not exceeding—pre-pandemic levels (**Table 1**).

**Table 1:** Annual cervical cancer cases and age-standardized incidence rates (ASIR), Mongolia, 2014–2024.

Year	Cases (n)	ASIR per 100,000	95% CI
2014	311	30.5	17.7–43.2
2015	360	36.3	23.5–49.0
2016	479	45.3	30.0–60.6
2017	365	33.6	20.7–46.4
2018	378	33.3	21.7–44.9
2019	328	27.6	17.2–37.9
2020	274	22.6	12.9–32.3
2021	210	16.5	8.3–24.7
2022	145	11.4	5.6–17.2
2023	508	38.7	26.7–50.8
2024	541	39.5	27.4–51.7

**Figure 1:**
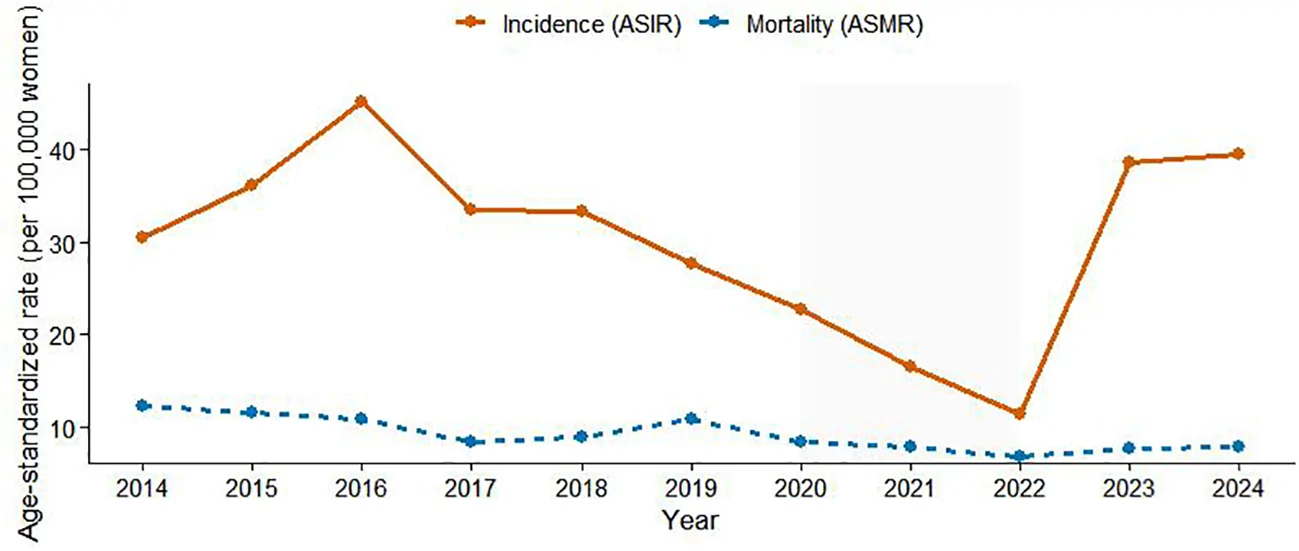
Trends in age-standardized incidence and mortality rates of cervical cancer in Mongolia, 2014–2024.

In contrast, age-standardized mortality rates (ASMR) demonstrated a more gradual and stable pattern over time (**Figure 1**). Mortality was highest at the beginning of the study period (approximately 12 per 100,000 women in 2014), declined through 2017, and showed a modest increase in 2019. Thereafter, ASMR decreased to its lowest level in 2022, followed by a slight increase in 2023–2024, although remaining below pre-2019 levels. Overall, temporal variation in mortality was considerably less pronounced than that observed for incidence.

### 3.3 Annual percent change in incidence

During the pre-pandemic period (2014–2019), ASIR showed a modest, non-significant decline (APC: −3.05%; 95% CI: −10.8 to 5.33). In contrast, a marked decline was observed during the pandemic period (2020–2022), with an APC of −29.4% (95% CI: −30.8 to −28.0). In the post-pandemic period (2023–2024), ASIR showed a slight increase; however, APC was not estimated due to the limited number of observations (two data points). Changes during this period are therefore described qualitatively and should be interpreted with caution (**Table 2**).

**Table 2:** Annual percent change (APC) in ASIR of cervical cancer by period, Mongolia, 2014–2024.

Period	APC (%)	95% CI
Pre-pandemic (2014-2019)	−3.05	−10.8 to 5.33
Pandemic (2020-2022)	−29.4	−30.8 to −28.0
Post-pandemic (2023-2024)	Not estimated	Not estimable

APC, annual percent change. Estimates were derived from log-linear regression of age-standardized incidence rates.

### 3.4 Mortality-to-incidence ratio

**Figure 2:**
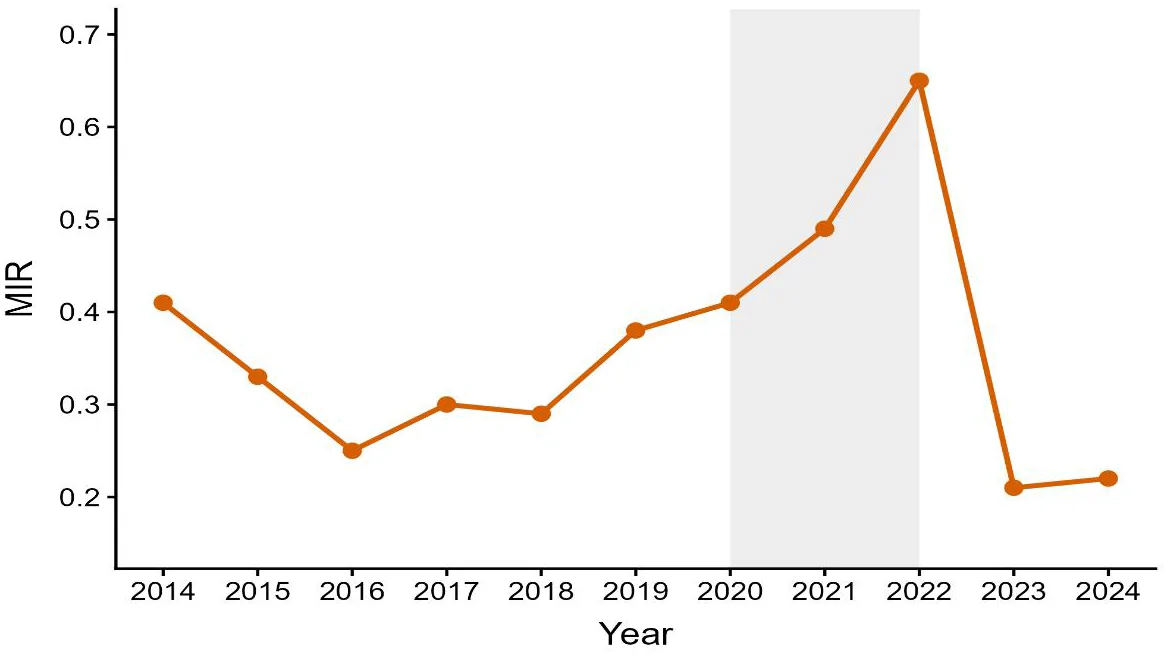
Temporal trends in the mortality-to-incidence ratio (MIR) for cervical cancer in Mongolia, 2014–2024.

The mortality-to-incidence ratio (MIR) declined from 0.41 in 2014 to 0.25 in 2016, followed by a gradual increase to 0.65 in 2022. Subsequently, MIR decreased sharply to 0.21 in 2023 and remained stable in 2024 (0.22). These changes correspond to periods of divergence between incidence and mortality, particularly during the pandemic years (**Table 3**). Temporal trends in MIR are illustrated in **Figure 2**.

**Table 3:** Age-standardized incidence, mortality, and mortality-to-incidence ratio (MIR), Mongolia, 2014–2024.

Year	ASIR	ASMR	MIR
2014	30.5	12.4	0.41
2015	36.3	11.8	0.33
2016	45.3	11.1	0.25
2017	33.6	10.2	0.30
2018	33.3	9.8	0.29
2019	27.6	10.5	0.38
2020	22.6	9.3	0.41
2021	16.5	8.1	0.49
2022	11.4	7.4	0.65
2023	38.7	8.2	0.21
2024	39.5	8.5	0.22

### 3.5 Segmented log-linear trend analysis of mortality (joinpoint-style)

Segmented log-linear regression identified a change point in mortality trends around 2022; however, the relatively large standard error of the estimated change point (2.54) indicates uncertainty in the exact timing due to the limited number of annual observations. Between 2014 and 2022, mortality declined significantly, with an annual percent change (APC) of −6.14% (95% CI: −9.20 to −2.97). In contrast, the trend during 2022–2024 showed a non-significant increase (APC: 3.65%; 95% CI: −27.93 to 49.06). Across the entire study period, mortality demonstrated a significant overall decline (AAPC: −4.76%; 95% CI: −7.08 to −2.37).

## 4 Discussion

This nationwide population-based study demonstrates that cervical cancer incidence in Mongolia is highly sensitive to short-term disruptions in health-system functioning, whereas mortality follows a more gradual and stable long-term pattern. The sharp decline in incidence during the COVID-19 pandemic, followed by a rapid rebound, highlights the extent to which cancer detection depends on the continuity of screening and diagnostic services (**Figure 1**).

The decline in incidence during the COVID-19 pandemic was particularly pronounced. Given the long natural history of cervical cancer, such rapid changes are unlikely to reflect a true reduction in disease occurrence (6, 9). Instead, these findings strongly suggest disruptions in screening programs, reduced access to diagnostic services, and changes in healthcare-seeking behavior during the pandemic. Similar patterns have been reported globally, where decreases in cancer diagnoses were largely attributed to reduced screening and delays in care (7, 10, 11). In Mongolia, preventive health services, including cervical cancer screening activities, were substantially reduced during 2020–2022 due to the reallocation of health system resources toward COVID-19 response and the implementation of movement restrictions, which likely contributed to reduced case detection (12, 13).

In contrast, mortality showed a overall downward trend over time and did not exhibit the abrupt changes observed in incidence (**Figure 1**). This distinction is important. While incidence is highly responsive to short-term changes in detection and diagnostic activity, mortality reflects longer-term processes, including access to treatment and survival outcomes. The absence of a corresponding decline in mortality during the pandemic is consistent with the interpretation that reduced detection, rather than true changes in disease occurrence, contributed to the observed fluctuations in incidence.

The mortality-to-incidence ratio (MIR) provides additional insight into these temporal patterns. As shown in **Figure 2**, MIR increased during the period of declining incidence and decreased following the recovery of health services. This pattern suggests that the observed fluctuations may be influenced, at least in part, by reduced screening and diagnostic activity rather than true changes in survival outcome. However, this interpretation should be considered alongside other plausible mechanisms. Disruptions in health-service delivery during the pandemic may have delayed diagnosis and treatment initiation, which could potentially contribute to shifts in stage at diagnosis; however, this could not be directly assessed in the present study. Similar patterns have been reported in other settings, where interruptions in cancer screening and care led to delayed diagnoses and concerns about future excess mortality (3, 10). In addition, reduced access to oncology services and interruptions in treatment continuity may have further compromised patient outcomes (7). Taken together, these findings suggest that the full impact of the pandemic on cervical cancer mortality may not be immediately observable but could emerge over time, reflecting a lagged effect of earlier disruptions in detection and care. Therefore, MIR trends should be interpreted with caution in the context of health-system shocks.

These results have important implications for the interpretation of routine cancer data and health-system evaluation. In settings where screening coverage is incomplete, incidence may reflect changes in diagnostic activity rather than true disease burden. The combined analysis of incidence, mortality, and MIR provides a more robust framework for distinguishing between changes driven by service disruptions and those reflecting underlying epidemiological patterns. In this context, the COVID-19 pandemic can be understood as a natural experiment that reveals the vulnerability of cancer detection systems to external shocks.

From a policy perspective, these findings underscore the importance of maintaining continuity of screening and diagnostic services during public health emergencies (14). Even temporary disruptions, such as those seen during 2020–2022, can lead to delayed diagnosis and may result in an increase in advanced-stage disease in the following years. Strengthening health-system resilience—by ensuring the continuity and integration of HPV vaccination, organized screening programs, effective referral pathways, and robust health information systems—will be essential for sustaining progress toward cervical cancer elimination and achieving WHO targets.

In a global context, the patterns observed in Mongolia are consistent with those reported in other low- and middle-income countries, where cervical cancer incidence and mortality remain elevated despite the availability of effective prevention strategies (2, 15, 16). In contrast, high-income countries have achieved substantial reductions in both incidence and mortality through organized screening programs and widespread HPV vaccination (15, 17, 18). The disruption in incidence observed during the COVID-19 period is also consistent with international evidence showing declines in cancer diagnoses due to reduced screening and delayed care (3, 7, 19). However, the relatively high mortality-to-incidence ratio in Mongolia suggests persistent challenges in early detection and access to timely treatment (20). These findings highlight the need to strengthen organized screening programs and improve equitable access to diagnostic and treatment services in line with WHO cervical cancer elimination targets (1).

These findings can be interpreted within a health-system performance framework, such as the Donabedian model (21), in which incidence reflects processes related to screening and diagnostic activity, while mortality reflects longer-term outcomes influenced by access to treatment, quality of care, and survival. The observed divergence between these indicators highlights the need to strengthen both early detection systems and treatment pathways to achieve sustained reductions in cervical cancer burden.

### 4.1 Strengths and limitations

This study has several important strengths. First, it is based on a nationwide, population-level dataset derived from Mongolia’s national health information system, ensuring comprehensive coverage of cervical cancer cases and minimizing selection bias. Second, the extended study period (2014–2024) allows for the assessment of long-term temporal trends and the identification of distinct phases, including pre-pandemic, pandemic, and post-pandemic periods. Third, the combined analysis of incidence, mortality, and the mortality-to-incidence ratio (MIR) provides a more nuanced interpretation of cancer trends, allowing differentiation between true epidemiological changes and variations driven by diagnostic activity. This integrative approach represents a key methodological strength of the study.

Several limitations should be acknowledged. First, information on individual screening participation and HPV vaccination status was not available, limiting our ability to directly assess the role of preventive services. Second, data on changes in stage at diagnosis over time were incomplete, making it difficult to determine whether observed trends in incidence and mortality reflect shifts in disease stage at presentation. Third, the mortality-to-incidence ratio (MIR) is an ecological indicator and does not capture individual-level survival, and should therefore be interpreted with caution. Furthermore, incomplete data on stage at diagnosis and treatment limit the ability to determine whether observed changes in MIR reflect differences in survival, delayed diagnosis, or variations in case detection. Fourth, the study relied on routinely collected administrative data, which may vary in completeness and accuracy over time, particularly during the pandemic period. Finally, some cases may not be captured if care was sought outside the public health system, which could lead to underestimation of incidence and mortality.

In conclusion, cervical cancer trends in Mongolia highlight the sensitivity of detection systems to health-service disruptions. The pandemic-related decline in incidence likely reflects reduced detection rather than true disease reduction. Maintaining continuity of screening and strengthening health-system resilience are essential to sustain progress toward cervical cancer control and WHO elimination targets.

## Data Availability

The datasets used and/or analyzed during the current study are not publicly available due to data protection regulations but are available from the corresponding author on reasonable request and with permission from the Center for Health Development, Mongolia.
