# Correction: Trends in cervical cancer incidence and mortality in Mongolia, 2014–2024: a population-based analysis of pandemic-related disruptions

**DOI:** 10.3389/fonc.2026.1993185

**Published:** 2026-10-09

**Authors:** Frontiers Production Office

**Affiliations:** Frontiers Media SA, Lausanne, Switzerland

**Keywords:** cervical cancer, COVID-19, health system resilience, incidence, Mongolia, mortality

The Editor was erroneously listed in the published article. The correct Editor is “L Gayani Tillekeratne”.

The original version of this article has been updated.

